# Randomized trial of the effect of intravenous paracetamol on inflammatory biomarkers and outcome in febrile critically ill adults

**DOI:** 10.1186/2008-2231-20-12

**Published:** 2012-08-28

**Authors:** Hooshyar Honarmand, Mohammad Abdollahi, Arezoo Ahmadi, Mohammad Reza Javadi, Mohammad Reza Khoshayand, Hamed Tabeefar, Sarah Mousavi, Laleh Mahmoudi, Mania Radfar, Atabak Najafi, Mojtaba Mojtahedzadeh

**Affiliations:** 1 Department of Clinical Pharmacy, Faculty of Pharmacy, Tehran University of Medical Sciences, Tehran, Iran; 2 Faculty of Pharmacy and Pharmaceutical Sciences Research Centre, Tehran University of Medical Sciences, Tehran, Iran; 3 Department of Anesthesiology and Critical Care Medicine, Sina Hospital, Faculty of Medicine, Tehran University of Medical Sciences, Tehran, Iran; 4 Department of Clinical Pharmacy, Faculty of Pharmacy, Tehran University of Medical Sciences and Health Services, Tehran, Iran; 5 Department of Clinical Pharmacy, Faculty of Pharmacy, Shiraz University of Medical Sciences, Shiraz, Iran; 6 Department of Clinical pharmacy, School of Pharmacy, Tehran University of Medical Sciences, 16 Azar Avenue, Tehran, Iran

**Keywords:** Fever, Systemic Inflammatory Response Syndrome (SIRS), Intensive Care Unit (ICU), Paracetamol, Cytokines

## Abstract

**Background and the purpose of the study:**

The febrile reaction is a complex response involving immunologic and other physiologic systems. Antipyretics are commonly used in critically ill patients with fever. We investigated the inflammatory responses following application of antipyretic therapy in febrile critically ill patients with Systemic Inflammatory Response Syndrome (SIRS).

**Patients and methods:**

In a prospective, randomized controlled study, critically ill patients with fever (T ≥ 38.3°C), SIRS diagnosed within 24 hours of Intensive Care Unit (ICU) admission and Acute Physiology and Chronic Health Evaluation II (APACHE II) score ≥10 were randomized into two groups. Upon appearance of fever, one group received intravenous paracetamol 650 mg every 6 hours for 10 days and other group received no treatment unless temperature reached 40°C. Body temperature, Acute Physiology and Chronic Health Evaluation II (APACHE II) and Sepsis-related Organ Failure Assessment (SOFA) scores, length of ICU stay, ICU mortality and infectious complications were recorded. Levels of Interleukin-1 alpha (IL-1_α_), IL-6, IL-10, Tumour Necrosis Factor alpha (TNF_α_) and High-Sensitive C-Reactive Protein (HS-CRP) were assessed at baseline and 2, 6 and 24 hours after intervention.

**Results and discussion:**

During a period of 15-month screening, 20 patients met the criteria and randomized to the control or paracetamol group. Body temperature decreased significantly in the paracetamol group (p = 0.004) and control group (p = 0.001) after 24 hours, but there was no significant difference between two groups at this time point (p = 0.649). Levels of IL-6 and IL-10 decreased significantly (p = 0.025 and p = 0.047, respectively) in the paracetamol group at 24 hours but this was not of statistical significance in control group. No patterns over time in each group or differences across two groups were found for HS-CRP, TNF_α_, and IL-1_α_ (p > 0.05). There were no differences regarding ICU length of stay, mortality and infectious complications between both groups.

**Conclusion:**

These results suggest that antipyretic therapy may not be indicated in all ICU patients. Allowing fever to take its natural course does not appear to have detrimental effects on critically ill patients with SIRS and may avoid unnecessary expenses.

## Introduction

Fever, defined as an increase in body temperature above 38.3°C (100.4°F), is among the most frequently detected abnormal physical signs in critically ill patients related either to SIRS or infections [1,2]. Fever is associated with increased length of stay in general ICU patients and poorer outcomes in certain patient groups such as those with Traumatic Brain Injury (TBI), Subarachnoid Hemorrhage (SAH) or pancreatitis [3,4]. Two prospective, observational studies of fever in the ICU suggested that fever is deleterious for ICU patients [5,6].

On the other hand, febrile response involves activation of immunologic, endocrinologic and other physiologic systems [7,8]. The response is generally beneficial, because such responses promote clearance of pathogenic microorganisms and hence improve outcome during infections [9].

Despite the contradictory effects of fever, antipyretic therapy including administration of antipyretic medications or application of external cooling is usually initiated [10]. The common justification for such therapy includes improved patient comfort, reduction in cardiovascular stress and avoidance of increased oxygen consumption [11].

Induction of fever is mediated by the release of pyrogenic cytokines such as TNF_α_, IL-1, IL-6 and interferons into the bloodstream in response to exogenous pyrogens. These cytokines play pivotal roles in inflammation and activation of the immune response. Extravagant activation of immune system with these inflammatory biomarkers can result in disastrous consequences like vascular collapse, shock and death [12-14]. Also increased temperature is known to induce changes in many of the effector cells of the immune response which could be harmful [15]. On the other hand, fever induces production of Heat Shock Proteins (HSP_s_) which subsequently reduces the levels of TNF_α_, IL-1, IL-6 and IL-10 [16]. Antipyretic therapy might mitigate the harmful effects of pyrogenic cytokines; however it may prevent the beneficial effects of fever.

The purpose of this study was to evaluate the effects of antipyretic therapy on inflammatory biomarkers in febrile critically ill patients with SIRS. We hypothesized that optimal application of antipyretic therapy improves clinical outcomes in critically ill patients with fever and SIRS.

## Methods

This prospective randomized clinical trial was conducted in the 12-bed general ICU of Sina Hospital affiliated to Tehran University of Medical Sciences (TUMS) in Tehran, Iran. The study received ethical approval from ethic committee of TUMS (Code No.: 9755) and written informed consent was obtained from patients or their kin.

During 15 months, all patients admitted to the ICU were screened for study eligibility and randomly assigned to one of two treatment groups. Computer-generated random number was utilized for randomization. Inclusion criteria were age ≥ 18 years, an APACHE II score ≥ 10, a core temperature ≥ 38.3°C accompanied by at least one other SIRS criterion (defined as the following conditions: heart rate > 90 beat/min, respiratory rate >20 breath/min or P_a_CO_2_ < 32 mmHg, white blood cells (WBC) > 12,000 cell/mm^3^, or < 4000 cell/mm^3^, or >10% immature [band] cells) diagnosed during the first 24 hours of ICU admission. Exclusion criteria were acute renal failure (defined as Cl_Cr_ < 50 ml/min or U/O < 0.5 ml/kg/h), liver dysfunction (defined as the presence of hepatic cirrhosis, hepatic encephalopathy or concentration of serum transaminases greater than third times the upper limit of normal range), SIRS diagnosed after 24 hours of ICU admission, history of seizure, stroke, or any acute brain injury (e.g. TBI), malignant hyperthermia (T ≥ 41°C), heat stroke, or Neuroleptic Malignant Syndrome (NMS), Ischemic Heart Disease (IHD), tympanic membrane inflammation or otitis (since body temperature is measured by infrared tympanic thermometer), age <18 years and pregnant or breast-feeding women.

Patients body temperatures were measured routinely (every 3 hours) by an infrared tympanic thermometer (Genius 2, USA). Upon appearance of fever, patients were randomized to receive a slow infusion of intravenous paracetamol (Apotel®, Uni Pharma, Greece) 650 mg every 6 hours for 10 days in the treatment group while in the control group, no antipyretic was administered unless the temperature reached 40°C.

Monitoring parameters including O_2_ saturation, non invasive blood pressure and central venous pressure, respiratory rate, heart rate and intake and output of each patient were recorded in the ICU flow sheets every 3 hours. Laboratory data including serum creatinine, Blood Urea Nitrogen (BUN), hemoglobin, platelet and WBC counts, activated Partial Thromboplastin Time (aPTT), Prothrombin Time (PT), International Normalization Ratio (INR), Erythrocyte Sedimentation Rate (ESR), albumin, sodium and potassium were also collected routinely and upon necessity.

Each group received standard treatments including early resuscitation within the first 6 hours of admission, appropriate diagnostic studies to ascertain causative organisms before starting antibiotics, early administration of broad-spectrum antibiotic therapy and reassessment of antibiotic therapy with microbiologic tests and clinical data to narrow coverage. Volume resuscitation was achieved with 0.9% normal saline and albumin for a target Central Venous Pressure (CVP) of 8–12 mmHg. After adequate fluid resuscitation, Mean Arterial Pressure (MAP) was kept between 60–90 mmHg, using vasopressors or inotrops if required. Mechanical ventilation were adjusted to maintain S_a_O_2_ > 95%, P_a_O_2_ > 60 mmHg and P_a_CO_2_ between 38 and 42 mmHg and appropriate analgesia and sedation were provided for all patients. Insulin treatment was administered to maintain glucose at < 200 mg/dl and standard prophylactic measures were made for Deep Vein Thrombosis (DVT) and Stress-Related Mucosal Damage (SRMD).

### Measurements

On the appearance of fever (hour 0) as a baseline, 2, 6, and 24 hours post administration of paracetamol or no antipyretic, venous blood samples were collected and centrifuged at 1500 rpm for 15 minutes to remove cells and cellular debris. The plasma was stored at −80°C until the time of analysis. Levels of IL-1_α_, IL-6, IL-10, TNF_α_ and High-Sensitive C-Reactive Protein (HS-CRP) were measured by commercially available enzyme-linked immunosorbent assay (ELISA) kits (Bender Med Systems Inc., Vienna, Austria) according to manufacturer’s instruction.

Sepsis-related Organ Failure Assessment (SOFA) score and APACHE II score were measured daily for 10 days. Length of ICU stay, ICU mortality, infectious complications and ventilator free days were recorded for all patients.

### Statistical analysis

Data are presented as mean ± standard error of mean. To assess differences between the treatment groups at each time point, the unpaired *t* test and the Mann–Whitney test were used for parametric and nonparametric variables, respectively. To assess differences between the time points in each treatment group, repeated-measure one-way analysis of variance and nonparametric analysis of variance (Friedman test) were used to analyze changes in temperature and levels of biomarkers. For the analysis of changes in variables with 2 time points (at baseline and 24 hours after), the paired *t* test and the Wilcoxon rank sum test were used for parametric and nonparametric variables, respectively. The Fisher exact test was used to compare ratios between the 2 groups. All tests reported a 2-sided *P* value with the level of significance set at 0.05.

## Results

### General characteristics and body temperature

Between November, 2009 and February, 2011, 307 consecutive patients admitted to the ICU were screened and 20 met criteria for enrollment. Ten patients were randomized to the control (no antipyretic) group and ten were randomized to the paracetamol group.

The patients in each group were similar at baseline regarding to age, gender, admission APACHE II and SOFA score (Table 1). The average of all temperatures, maximum temperature of all days, incidence rates of infection (based on culture results), ICU length of stay, ventilator free days, and ICU mortality were similar for both groups.

**Table 1:** Demographic characteristics of febrile patients

	**No antipyretic (n = 10)**	**Paracetamol (n = 10)**	**p Value**
**Age (year)**	45.4 ± 6.67	49.5 ± 5.37	0.638
**Male/Female (No.)**	6/4	8/2	>0.05
**APACHE II score**	14.5 ± 1.00	14.9 ± 0.81	0.760
**SOFA score**	5.4 ± 0.89	5.7 ± 1.20	0.843
**T**_**max**_**(°C)**	38.81 ± 0.15	38.83 ± 0.14	0.921
**Mean of all temperatures (°C)**	37.39 ± 0.14	37.32 ± 0.18	0.762
**Proven infection (No.)**	5	4	>0.05
**ICU stay (day)**	22.1 ± 3.43	23.6 ± 4.32	0.789
**Ventilator free days**	33.3%	35.7%	0.887
**ICU mortality (No.)**	3	2	>0.05

p values <0.05 considered as significant. Data shown are mean ± SE.

**Figure 1:**
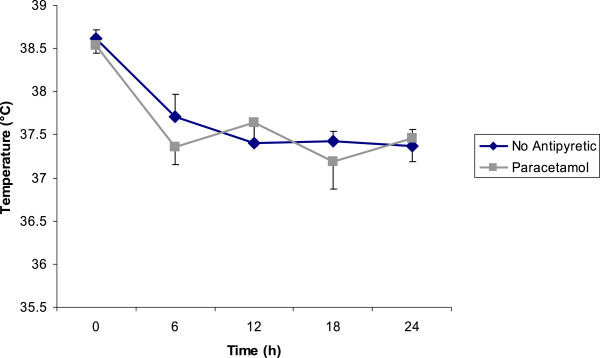
**Evolution of body temperature after paracetamol and no antipyretic treatment.** Temperature well decreased in both groups after 24 hours (p < 0.05); but differences between two groups were not significant (p > 0.05). Figure shows mean temperatures ± SE.

The mean temperature decreased from 38.53 ± 0.08°C to 37.46 ± 0.27°C (p = 0.004) in the treatment group and from 38.62 ± 0.10°C to 37.37 ± 0.19°C (p = 0.001) in the group without treatment at 24 hours (Figure 1). There was no significant difference between two groups at this time point (p = 0.649).

In our study intravenous paracetamol was well tolerated and no adverse effects regarding hepatotoxicity –defined as levels of serum transaminases more than third times the upper limit of normal range- were seen in the treatment arm.

### Biomarkers assay

HS-CRP, IL-6 and IL-10 were highly elevated at baseline and after 24 hours compared to normal concentrations in two groups (Table 2).

**Table 2:** **Cytokine concentrations at baseline and 24 hours after intervention (t**_**24**_**)**

	**No Antipyretic**			**Paracetamol**		
	**Baseline**	**t**_**24**_	**p Value**	**Baseline**	**t**_**24**_	**P Value**
**HS-CRP (mg/l)**	11.69 ± 3.00	12.18 ± 3.40	0.753	15.26 ± 3.70	12.13 ± 3.46	0.236
**TNF**_**α**_**(pg/ml)**	5.16 ± 1.35	4.7 ± 0.62	0.285	4.68 ± 1.32	3.8 ± 1.05	0.401
**IL-1**_**α**_**(pg/ml)**	1.33 ± 0.63	1.27 ± 0.56	0.674	1.57 ± 1.26	1.17 ± 0.87	0.397
**IL-6 (pg/ml)**	62.33 ± 22.58	47.96 ± 18.74	0.889	88.79 ± 23.15	30.12 ± 8.20	0.025
**IL-10 (pg/ml)**	73.85 ± 35.76	58 ± 20.39	0.878	93.64 ± 46.60	48.36 ± 23.35	0.037

p values <0.05 considered as significant.

Data shown are mean ± SE.

**Figure 2:**
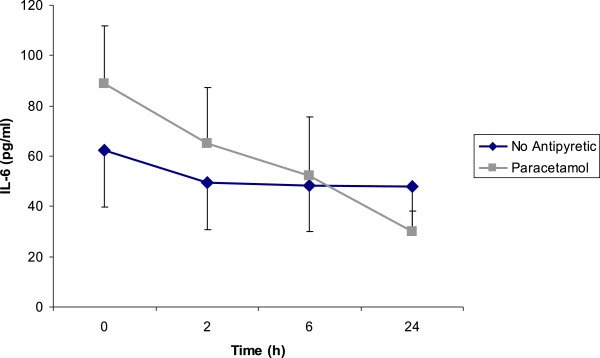
**Interleukin 6 levels in paracetamol and no antipyretic groups.** IL-6 decreased significantly in paracetamol group (p < 0.05); but not in the no treatment arm. However, differences between two groups were not significant (p > 0.05). Figure shows mean IL-6 levels ± SE.

**Figure 3:**
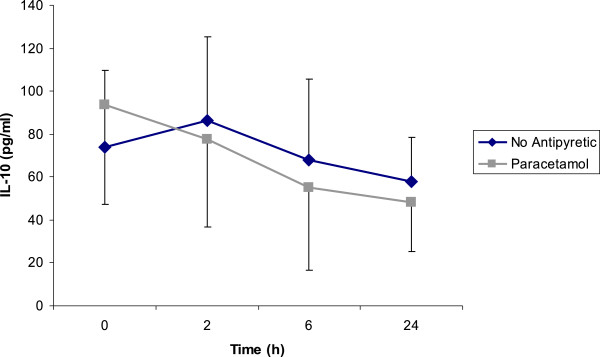
**Interleukin 10 levels in paracetamol and no antipyretic groups.** IL-10 decreased significantly in paracetamol group (p < 0.05); but not in the no treatment arm. However, differences between two groups were not significant (p > 0.05). Figure shows mean IL-10 levels ± SE.

IL-6 and IL-10 decreased significantly (p = 0.025 and p = 0.037, respectively) in the paracetamol group at 24 hours but this was not of statistical significance in control group (p = 0.889 and p = 0.878, respectively) (Figures 2 and 3). However; at none of the time points (0, 2, 6, and 24 h), there were no significant differences between two groups (p > 0.05). No patterns over time in each group or differences across two groups were found for HS-CRP, TNF_α_, and IL-1_α_ (p > 0.05).

## Discussion

The present findings show that intravenous paracetamol has no effect on fever and fever is resolved spontaneously without any intervention. Meanwhile treatment of fever had no effect on length of ICU stay, incidence rates of infection, mechanical ventilation dependency and ICU mortality.

Fever is characterized by an elevation of body temperature above normal variation due to an altered hypothalamic set point. It has been shown that fever is the product of endogenous pyrogens such as IL-1, IL-6 and TNF_α_[1]. There are still contradictory results about beneficial or deleterious effects of fever. Considerable amount of evidence shows that fever is an important defense mechanism which contributes to the host’s ability to resist infections [1,17]. Temperature in the range of the usual fever renders host defenses more active and many pathogens more susceptible to these defenses [18]. On the other hand, potential adverse consequences of fever such as increased metabolic demand may be extremely detrimental in certain patient’s population such as those with neurologic and cardiovascular conditions and make intervention necessary [2-4]. Two prospective, observational studies in critically ill febrile patients showed that fever is harmful for ICU patients. Circiumaru et al. [5] in a research on 100 patients found that prolonged fever more than five days was related to poorer outcome. Barie et al. [6] studied 626 febrile patients and found that peak temperature was the most powerful predictor of mortality.

Laupland et al. [3] in another retrospective cohort study on 20466 patients showed that the incidence of fever was higher in trauma/neuro patients, males, younger patients and those with admission APACHE II score less than 25. They also found that fever alone (T ≥ 38.3°C) was not associated with increased risk of death (13% vs. 12%; p = 0.08), but that high fever (T ≥ 39.5°C) was associated with significantly increased ICU mortality (20.3% vs. 12%, p < 0.0001). However, none of these studies evaluated the effects of antipyretic therapy on patients’ outcome.

However results of randomized trials with antipyretic show that treatment of fever may not be indicated in all ICU patients. In a randomized prospective study of antipyretics in 38 surgical ICU patients without neurotrauma or severe hypoxemia and with SIRS and fever, Gozzoli et al. [19] found no significant differences in recurrence of fever, incidence of infection, antibiotic therapy, ICU or hospital length of stay, and mortality. Our results also confirmed that antipyretic therapy had no effect on patients’ outcome. In another prospective study, Schulman et al. [20] randomized 82 critically ill patients into aggressive (44 patients) or permissive (38 patients) groups. The aggressive group received acetaminophen 650 mg every 6 h for temperature of > 38.5°C. The permissive group received no treatment for temperature of >38.5°C, but instead had treatment initiated at temperature of >40°C. No significant differences regarding incidence rates of infection were noted between two groups. There were seven deaths in the aggressive group and only one death in the permissive group (p = 0.06). The trial was ceased after the first interim analysis due to the mortality difference and it was thought that aggressively treating fever in critically ill patients may lead to a higher mortality.

Gazzoli et al. [21] in an open-label randomized trial in 30 mechanically ventilated surgical ICU patients, investigated the metabolic, hemodynamic and inflammatory responses of pharmacologic (intravenous propacetamol or metamizol) and physical therapy (external cooling) to reduce body temperature. They concluded that both treatments (pharmacologic and external cooling) equally reduced temperature. The inflammatory responses (IL-6, IL-8, TNF_α_ and CRP) were not influenced by three antipyretic treatments, although IL-6 tended to decrease over time in the metamizol arm. Similarly in our study levels of HS-CRP and cytokines (IL-1_α_ and TNF_α_) were not statistically different between two groups at any of the time points during intervention, but levels of IL-6 and IL-10 decreased significantly in paracetamol group at 24 hours and this was not of significant difference in group without treatment. However, no differences regarding these cytokines were seen between two arms. Some studies indicate the correlation between early levels of IL-6 and IL-10 and Injury Severity Score (ISS), development of Multi Organ Dysfunction Syndrome (MODS) and mortality in polytraumatized ICU patients [22-25]. Since IL-10 is an anti-inflammatory cytokine, increased levels could be attributed to a compensatory reaction to hamper deleterious overinflammation. In our trial, paracetamol significantly reduced both levels of IL-6 and 10, so its effect on inflammatory response remains unclear. Despite the fact that pyrogenic cytokines mediate the potential role in febrile response and physiologic abnormalities due to certain infections [15,26,27], in our study along with previous reports [22,23], interventions to decrease the level of these biomarkers was not beneficial in patients’ outcome.

## Conclusion

Fever is triggered by the release of various cytokines, notably IL-1, IL-6 and TNF_α_ which in turn can affect the immune response and defense mechanisms in a complex way. Antipyretics might mitigate the harmful effects of pyrogenic cytokines; however they may also hurdle the beneficial effects of fever. The present study, in addition to previous reports, suggests that antipyretic therapy may not be justified in all ICU patients, especially those without malignant hyperthermia, neurotrauma or other acute neurological disorders. Until sufficient data from large randomized clinical trials is available, allowing fever to take its natural pathway does not seem to have detrimental effects on all critically ill patients with SIRS and may avoid unnecessary expenses.

## Competing interests

The authors declare that they have no competing interests.

## Authors’ contributions

HH designed the study, selected the patients, obtained, analyzed and interpreted the data, and drafted and revised the manuscript. MA conceived and designed the study, and revised the manuscript. AA designed the study, selected the patients, and obtained the data. MRJ designed the study. MRK carried out statistical analysis, and interpreted the data. HT selected the patients, and obtained the data. SM drafted and revised the manuscript. LM obtained the data, and drafted the manuscript. MR designed the study. AN selected the patients. MM conceived and designed the study, selected the patients, interpreted the data, and revised the manuscript. All authors read and approved the final manuscript.
